# High Macromolecular Crowding in Liposomes from Microfluidics

**DOI:** 10.1002/advs.202201169

**Published:** 2022-07-29

**Authors:** Luis P. B. Guerzoni, André V. C. de Goes, Milara Kalacheva, Jakub Haduła, Matthias Mork, Laura De Laporte, Arnold J. Boersma

**Affiliations:** ^1^ DWI‐Leibniz Institute for Interactive Materials Forckenbeckstrasse 50 52074 Aachen Germany; ^2^ Institute of Technical and Macromolecular Chemistry RWTH Aachen University Worringerweg 1 52074 Aachen Germany; ^3^ Department Advanced Materials for Biomedicine Institute of Applied Medical Engineering University Hospital RWTH Aachen Forckenbeckstrasse 55 52074 Aachen Germany

**Keywords:** FRET sensors, macromolecular crowding, microfluidics, sartificial cells

## Abstract

The intracellular environment is crowded with macromolecules that influence biochemical equilibria and biomacromolecule diffusion. The incorporation of such crowding in synthetic cells would be needed to mimic the biochemistry of living cells. However, only a few methods provide crowded artificial cells, moreover providing cells with either heterogeneous size and composition or containing a significant oil fraction. Therefore, a method that generates monodisperse liposomes with minimal oil content and tunable macromolecular crowding using polydimethylsiloxane (PDMS)‐based microfluidics is presented. Lipid stabilized water‐in‐oil‐in‐water emulsions that are stable for at least several months and with a high macromolecular crowder concentration that can be controlled with the external osmolality are formed. A crucial feature is that the oil phase can be removed using high flow conditions at any point after production, providing the highly crowded liposomes. Genetically encoded macromolecular crowding sensors show that the high level of macromolecular crowding in the emulsions is fully retained throughout the generation of minimal‐oil lipid bilayers. This modular and robust platform will serve the study of biochemistry under physiologically relevant crowding conditions.

## Graphical abstract

A method to achieve monodisperse liposomes with high internal macromolecular crowding is presented. Double emulsions obtained from microfluidics are highly robust and reproducible and can be shrunk to desired crowding through osmosis. Crucially, the oil phase can be removed at any point using a centrifugation step to yield liposomes, while maintaining crowding as verified with a crowding sensor.

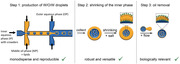

## 1 Introduction

Cells are the basic building block of life and a cornerstone of fields such as biotechnology, synthetic biology, medicine, drug delivery, pharmaceuticals, biosensors, and bioremediation. However, for practical applications and as a study object, most living cells are fragile, are biochemically highly complex, and undergo facile apoptosis. To overcome these issues, scientists construct synthetic microcompartments as artificial cells.^[^ ^1^ ^]^ Artificial cells are defined as cell‐like structures that display at least some of the properties found in native cells, such as the ability to self‐maintain, proliferate, evolve, and die. Top‐down construction of synthetic cells removes components from living cells,^[^ ^2^ ^]^ while bottom‐up synthesis consists of assembling molecular components into an artificial cell.^[^ ^3^ ^]^ The main advantage of bottom‐up assembly compared to living cells is the high control over the composition, which allows better study or use of its biochemistry. Of these components, the lipid bilayer is an important asset. The biomembranes localize biochemical pathways, protecting its biochemistry from the environment and establishing chemical gradients and selective permeation. Therefore, a lipid membrane is a highly desirable component in artificial cells.

Besides encapsulation with a membrane, a striking feature of living cells is that they are highly crowded with macromolecules (50–400 mg mL^–1^),^[^ ^4^ ^]^ influencing the conformation, assembly formation, and diffusion of the different biomacromolecules.^[^ ^5^ ^]^ Mimicking physiological crowding in artificial cells would present a significant advance in understanding the behavior of biomacromolecules since artificial cells allow a higher level of control over their contents, resulting in the ability to tune macromolecular crowding and subsequent observation of the effect of crowding on a specific protein. Moreover, a crowded artificial cell takes into account the interplay of crowding with the limited absolute number of molecules and the presentation of a lipid membrane for nonspecific adsorption. Crowding has thus been introduced in giant unilamellar vesicles with classical batch‐based processes,^[^ ^6^ ^]^ albeit these methods generally provide a broad size distribution of vesicles. Nonetheless, this work showed that vesicles can contain elevated concentrations of synthetic crowders and cell lysates. However, concentrating the content of these vesicles requires skill, as the lipid bilayer is sensitive to osmotic stress, generating transient lipid pores in the membrane that spill content into the surrounding media.^[^ ^6a^ ^]^

Precise control over reproducible production, monodispersity, and high encapsulation efficiency can be achieved with microfluidics.^[^ ^5^ ^]^ In recent years, double‐emulsion droplet microfluidics emerged as an outstanding technique for droplet manipulation at the micrometer scale.^[^ ^7^ ^]^ It allows for the generation of well‐defined and monodisperse microcompartments that encapsulate a defined set of functional biomolecules.^[^ ^1^, ^8^ ^]^ For example, water‐in‐oil‐in‐water (W/O/W) droplets were generated with biomolecular condensates in the inner aqueous core, and with phospholipids in the middle oil phase using PDMS‐based microfluidics.^[^ ^9^ ^]^ Notably, most of the oily phase spontaneously detached from the aqueous core with 15% glycerol in the medium.^[^ ^9^ ^]^ Similarly, 28% ethanol allowed removal of the oleic acid phase over three days.^[^ ^10^ ^]^ Recently, it was shown that high flow in microfluidics could also reduce the oil phase to generate liposomes without the need for surfactants or other additives.^[^ ^11^ ^]^ While these examples did not provide crowded artificial cells, the Huck group developed two approaches for crowded artificial cells using microfluidics. The first example was based on a water in oil emulsion stabilized by block copolymers, which allowed the study of transcription and translation in a crowded environment.^[^ ^12^ ^]^ In the second example, capillary‐based microfluidics was used to fabricate W/O/W emulsions where most of the oil resided in a lipid pocket.^[^ ^13^ ^]^ These compartments could be shrunk and crowded upon applying a hyperosmotic upshift to the outer phase. It was hypothesized that excess lipid generated during shrinkage would be buffered by uptake in a large oil pocket in the membrane. These artificial cells allowed the study of transcription and translation under crowded conditions, showing the high sensitivity of these biochemical reactions to the crowder content.^[^ ^14^ ^]^

**Figure 1:**
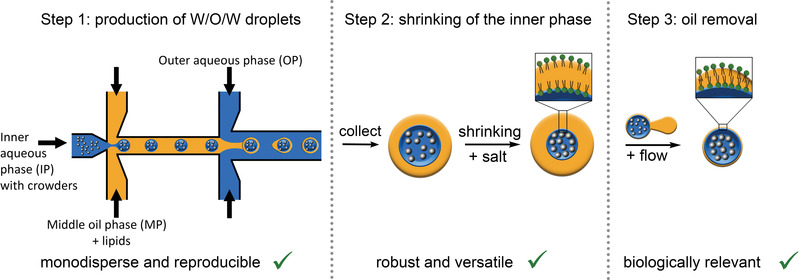
Stepwise approach to achieve crowded liposomes. PDMS‐based microfluidics provides monodisperse and reproducible W/O/W droplets that are highly stable and can be manipulated. Using a separate flow step allows removing the oil layer from the manipulated droplets to provide liposomes.

In this work, we aimed to construct crowded artificial cells by i) PDMS‐based microfluidics to achieve high reproducibility, monodispersity, robustness, and versatility, ii) and in the absence of an oil pocket that may absorb small molecules or hamper membrane proteins. We thus present liposomes with minimal oil content that contain high levels of crowding as verified with a macromolecular crowding sensor and with the PDMS‐based droplet microfluidics intrinsic monodispersity and reproducibility. A crucial step in the manufacturing is the realization of a two‐step process (**Figure** **1**), where first a double emulsion template is constructed that is highly stable and can be manipulated at will, after which the oil layer can be removed using high flow conditions to generate liposomes. We thus combine the advantages of previous approaches, which are the stability of double emulsion droplets to obtain exceptional high levels of crowding and the pinch‐off capability of the oil layer to achieve a liposome.

## 2 Results and Discussion

### 2.1 Lipid Stabilized Water‐Oil‐Water Emulsions from PDMS‐Based Microfluidics

We first designed a microfluidic chip to produce lipid stabilized W/O/W double emulsion droplets (Figure 1A,B, Figures S1 and S2, Supporting Information). To achieve W/O/W double emulsion droplets within a single device, a hydrophobic and a hydrophilic section are needed.^[^ ^15^ ^]^ For this, we applied a sequential layer‐by‐layer deposition of oppositely charged polyelectrolytes following a recently developed protocol, with some modifications (see Experimental Section).^[^ ^10^ ^]^ We incorporated 50 mg mL^–1^ Pluronic F‐127 in the outer aqueous phase (OP) as a non‐ionic copolymer stabilizer, which would not interfere with the biomolecules in the inner solution. In the OP and the inner aqueous phase (IP), a 10 mm sodium phosphate (NaPi) buffer, pH 7.4, was used to keep neutral pH. The inner phase contained the macromolecular crowder at 80 mg mL^–1^, which was either Ficoll PM70, a sugar‐based branched polymer of 70 kD, or bovine serum albumin (BSA), both frequently used in macromolecular crowding studies.^[^ ^16^ ^]^ We first chose n‐octanol as the middle oil phase (MP), as this oil has been shown to spontaneously separate from double emulsion droplets during production in the presence of 15% glycerol.^[^ ^9^ ^]^ In contrast to previous work on crowded vesicles,^[^ ^13^ ^]^ the MP did not contain surfactants, only the natural lipids [(2R)‐3‐Hexadecanoyloxy‐2‐[(Z)‐octadec‐9‐enoyl]oxypropyl] 2‐(trimethylazaniumyl)ethyl phosphate (POPC), [(2R)‐1‐[2,3‐dihydroxypropoxy(hydroxy)phosphoryl]oxy‐3‐hexadecanoyloxypropan‐2‐yl] (Z)‐octadec‐9‐enoate (POPG) and cholesterol, which is a common mixture for generating stable liposomes in batch‐based techniques.

**Figure 2:**
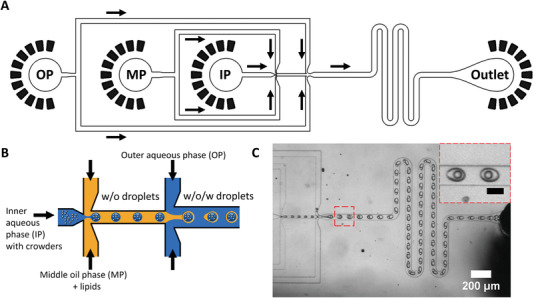
Double emulsion production in PDMS‐based microfluidics. A) Design of the microfluidic device using a double junction design. The junctions contain a flow‐focusing architecture. Serpentine‐shaped buffering channels are included to improve mixing within the double emulsions. Flow directions are indicated with black arrows. B) Cartoon of the principle to generate W/O/W droplets using two junctions, where the first junction mixes the MP with the IP to generate single W/O emulsions, which are mixed with the OP at the second junction to provide W/O/W emulsions. C) Optical microscopy image of continuous production of W/O/W emulsions using the architecture of panel A. Inset black scale bar is 50 µm. The MP consists of n‐octanol with 5 mg mL^–1^ of POPC:POPG:Cholesterol 8.5/1.0/0.5, and 0.1% DiD. The IP contains 10 mm NaPi, 80 mg mL^–1^ BSA, pH 7.4, the OP contains 50 mg mL^–1^ Pluronic F‐127 + 10 mM NaPi, pH 7.4.

**Figure 3:**
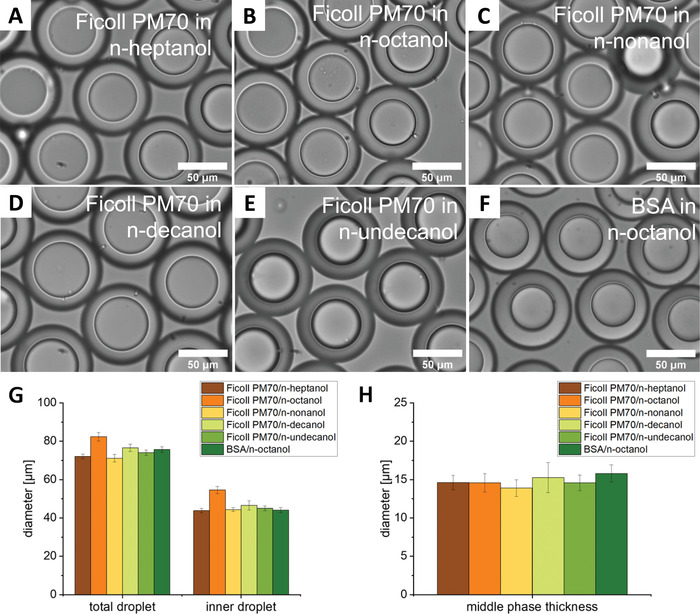
Brightfield images of W/O/W emulsions with an IP containing 80 mg mL^–1^ Ficoll 70 and an MP consisting of A) n‐heptanol, B) n‐octanol, C) n‐nonanol, D) n‐decanol, E) n‐undecanol. F) W/O/W droplet containing 80 mg mL^–1^ BSA as IP, and n‐octanol as MP. G) Average diameters of the entire emulsion “total droplet” and of the IP “inner droplet” show little dependence on the composition. H) Corresponding thickness of the MP. All the MPs contain 5 mg mL^–1^ of POPC:POPG:cholesterol 8.5/1.0/0.5. The IP contains 10 mm NaPi, pH 7.4, the OP contains 50 mg mL^–1^ Pluronic F‐127 + 10 mM NaPi, pH 7.4. Data are mean values (*n* = 10 emulsions). Error bars are SD.

We first optimized the composition of the MP to form stable emulsions. We found that 5 mg mL^–1^ of the blend POPC:POPG:cholesterol in a mass ratio of 8.5/1.0/0.5 in n‐octanol provided stable double emulsions (**Figures** **2**C and **3**B, Video S1, Supporting Information). We used 80 mg mL^–1^ Ficoll as crowder in the IP in these experiments. Further increasing the lipid concentration led to multiple smaller satellite droplets, possibly to maximize the water–oil surface area. Experiment‐dependent small satellite droplets occasionally form, likely due to a combination of specific flow rates and channel dimensions.^[^ ^17^ ^]^ We did not observe the spontaneous separation of the n‐octanol as shown before,^[^ ^9^ ^]^ which may be due to the absence of glycerol and lower flow rates in our device. We did not add glycerol as it is permeable over membranes and would inhibit osmotic shrinkage, and may affect some biochemical reactions. Because n‐octanol has a high water partitioning coefficient, which may interfere with biochemical reactions in the vesicles, we increased the size of the alcohol's alkyl chain, as each –CH_2_– added decreases the water solubility roughly by an order of magnitude. We tested n‐heptanol, n‐octanol, n‐nonanol, n‐decanol, and n‐undecanol, and found that the alkyl chain length had no significant influence on the stability of the double emulsions (Figure 3A–E). Alcohols with even longer alkyl chains are not liquid at room temperature and were not tested. Replacing Ficoll for bovine serum albumin (BSA) at the same concentration gave virtually the same emulsions (Figure 3F). The corresponding droplet diameters varied only slightly between these experiments, most likely due to experimental variation (Figure 3G,H). The average outer diameter was ≈70 µm, and the inner was ≈45 µm. To verify that PDMS does not significantly swell when coming into contact with these organic solvents, which would decrease the dimensions of the microfluidic channels and result in reproducibility issues and device breakage, we determined the swelling properties of PDMS in contact with these alcohols. We found a maximum of 2% swelling after 120 min incubation in PDMS, with the smaller chain alcohols providing a somewhat higher swelling than the longer chain alcohols (Figure S3, Supporting Information). Hence, swelling is practically negligible, and these alcohols are compatible with PDMS‐based microfluidic devices.

### 2.2 W/O/W Emulsions Are Highly Stable and Can Be Shrunk to Achieve High Crowding

**Figure 4:**
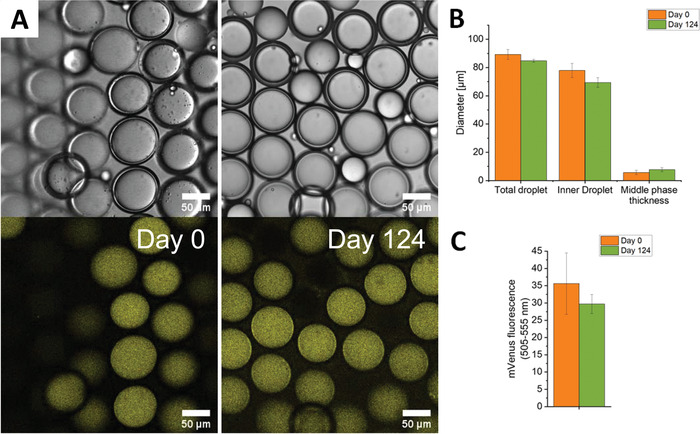
W/O/W emulsions containing 150 mg mL^–1^ Dextran 40 kD are highly stable in time. A) Top: brightfield images, bottom: fluorescence confocal images of an mVenus‐mCherry construct containing emulsions. Excited at 488 nm, emission at 505–555 nm. B) Stability of the average diameter of the total and inner droplet and that of the thickness of the middle phase over 124 days. C) Corresponding fluorescence emission in the mVenus channel. The MPs contain 5 mg mL^–1^ of POPC:POPG:cholesterol 8.5/1.0/0.5 in n‐octanol. The IP contains 10 mm NaPi, pH 7.4, 150 mg mL^–1^ Ficoll PM70, the OP contains 50 mg mL^–1^ Pluronic F‐127 + 10 mM NaPi, pH 7.4. Data are mean values (*n* = 20 emulsions). Error bars are SD.

We assessed the stability of the emulsions by incorporating fluorescent proteins together with either Ficoll PM70 or Dextran 40 kD as crowder at 150 mg mL^–1^ and a control population without crowder. The emulsions were imaged directly after production, and the remainder was stored at room temperate in a sealed container to prevent water evaporation. We imaged the emulsions again after 18 weeks and found that the dextran‐containing emulsions had similar dimensions and fluorescent intensity (**Figure** **4**), while the Ficoll crowded vesicles showed a minor decrease in size (Figure S4, Supporting Information). The crowder is required for high stability because the inner phase shrank considerably without the crowder (Figure S5, Supporting Information). Likely, crowders retain water by maintaining or increasing the osmotic pressure in the IP. Hence, when the droplets contain crowding, they do not leak and are highly stable for at least 18 weeks, and most likely a multitude of that timeframe.

**Figure 5:**
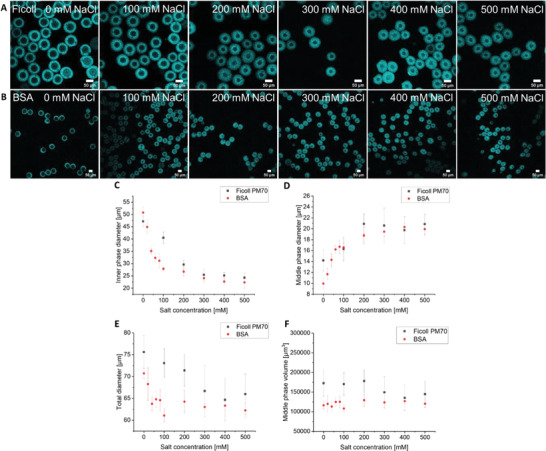
W/O/W shrinkage through osmosis. A) Fluorescence confocal microscopy images of the W/O/W emulsions containing an 80 mg mL^–1^ starting concentration of Ficoll PM70 and the subsequent shrinkage upon titration of NaCl to the medium. B) As in panel (A), with 80 mg mL^–1^ BSA as crowder. C) Dependence of the IP diameter on the external NaCl concentration for the Ficoll PM70 and the BSA crowded vesicles showing a NaCl‐dependent shrinking. D) Corresponding diameter of the oil phase showing an increase in thickness with NaCl as the inner phase shrinks. E) Diameter of the entire droplet as a function of the NaCl concentration showing a less strong effect. F) The volume of the oil phase as a function of NaCl concentration shows that the MP stays intact during shrinkage. The MP consists of n‐octanol with 5 mg mL^–1^ of POPC:POPG:cholesterol 8.5/1.0/0.5. The IP contains 10 mm NaPi, pH 7.4, the OP contains 50 mg mL^–1^ Pluronic F‐127 + 10 mM NaPi, pH 7.4. The MP is visualized using DiD. Data are mean values (*n* = 10 emulsions). Error bars are SD.

To achieve high crowding levels, the double emulsions containing Ficoll PM70 or BSA were exposed to increasing levels of NaCl after production (**Figure** **5**A,B, Figure S6, Supporting Information). NaCl does not permeate bilayers, which leads to an osmotic pressure difference over the MP, inducing a water flux until isoosmolality over the membrane is restored. Direct manufacturing of emulsions with high internal crowding was precluded by the exceptional high viscosity of crowded solutions. We added DiIC18(5); 1,1’‐dioctadecyl‐3,3,3’,3’‐tetramethylindodicarbocyanine, 4‐chlorobenzenesulfonate salt (DiD) to the MP as a lipid dye for better visualization. As expected, the aqueous IP decreased considerably in size from 45 to 25 µm in diameter due to water diffusion through the MP to the OP, both in the case of Ficoll PM70 and BSA (Figure 5C). This corresponds to a maximum 8‐fold decrease in the IP volume (Figure S7, Supporting Information), corresponding to ≈650 mg mL^–1^ BSA, assuming no crowder loss. Indeed, we see an increase in fluorescence when incorporating a fluorescent crowding sensor (see below) during osmosis‐induced shrinkage (Figure S8, Supporting Information). We find that the emulsions containing BSA shrink more at lower NaCl concentrations than the Ficoll PM70 crowded emulsions. Such difference could be due to the colligative properties of the crowders that change the internal osmolality at a specific crowder concentration. Throughout the experiment, the thickness of the MP increases (Figure 5D) due to the reduction in the volume of the IP. The unchanged volume of the MP (Figure 5F) shows that octanol is neither lost nor gained throughout the manipulation.

Hence, the double emulsion formed with lipids and higher alcohols is exceptionally stable, allowing measurements over four months, and osmosis facilitated shrinkage to achieve very high crowding levels.

### 2.3 Removal of the Oily Middle Phase with a Flow to Obtain Liposomes

**Figure 6:**
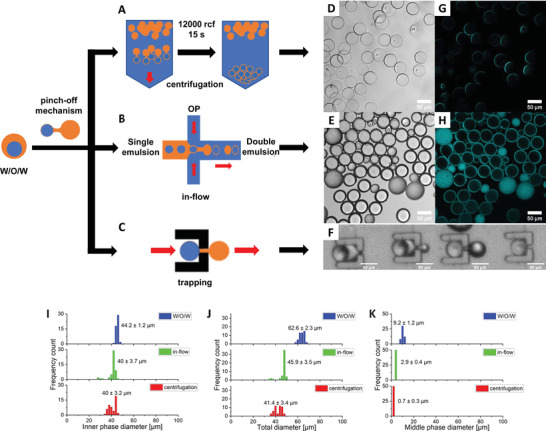
High‐flow approaches allow MP removal from W/O/W emulsions. Schematic of three approaches applied here to remove the MP from W/O/W emulsions, all based on the density difference between the aqueous and oil phase leading to a putative pinch‐mechanism induced by flow. A) The MP oil layer is retained at the air/water interphase upon spinning down the W/O/W emulsions. B) High flow directly during production. C) Trapping W/O/W emulsions. D–H) Brightfield and fluorescence microscopy images of the resulting liposomes. MP stained with DiD. I–K) Histograms of the IP diameter, the total diameter, and the MP diameter show the size distribution of the liposomes before and after manipulation. The MP diameter after liposome formation is an estimate as it is below the resolution of a regular confocal fluorescence microscope. Emulsions contain 80 mg mL^–1^ BSA as crowder in the IP and n‐octanol as MP. The emulsion in the trapping device contains 80 mg mL^–1^ Ficoll PM70. Depicted values are mean ± SD (*n* = 50 emulsions).

While the double emulsions are clearly highly stable and versatile, we aimed to construct liposomes with minimal oil content, as oil could otherwise interfere with biochemical experiments. We observed that in contrast to n‐octanol, n‐undecanol resulted in a spontaneous dewetting of the oil phase after incubation of the double emulsion at room temperature over 10 days (Figure S9, Supporting Information). This is perhaps caused by the less polar nature of n‐undecanol, causing the MP and the IP to form two separate droplets. Hence, agents such as glycerol and ethanol may not be needed to assist in dewetting when using n‐undecanol instead of n‐octanol. However, to achieve a controllable and on‐demand dewetting process, we enforced dewetting by placing the double emulsion under high flow, making use of the lower density of the oil phase (**Figure** **6**B, Video S2, Supporting Information). The oil phase is less dense and lags behind the liposome with a higher density. Indeed, previous research confirmed that the lipid layer could be decreased by separating the oil layer from W/O/W emulsions during production under high flow.^[^ ^11^ ^]^ We applied high flow conditions during the emulsion production and followed the MP diameter with a DiD fluorescent stain. We found a reduction in the size of the MP diameter from 9.0 ± 1.2 to 2.9 ± 0.3 µm. These vesicles have the same monodispersity as the double emulsions produced at lower flow rates (Figure 6E,H,I–K).

To separate the production and the oil removal step so that liposomes can be made more crowded by osmosis, we captured the double emulsions in engineered wells in a second device and left them under high flow (Figure 6C, Figure S10, Video S3, Supporting Information). This would allow immobilization and bilayer formation in a single device and provide a high flow difference between the stationary liposome and the separating oil droplet. We engineered the wells to contain a hole that would allow the oil to escape. Indeed, the wells allowed capturing the W/O/W emulsions, and vesicles were formed via the intended pathway. However, the experiment‐dependent yield of the process and its complexity made it impractical for further applications. Nevertheless, it reemphasizes that high flow can induce the physical separation of the MP and the lipid bilayer.

To achieve a high flow in a postproduction step that first allowed osmotic manipulation of the stable emulsions followed by a fast and simple oil removal procedure, we centrifuged the W/O/W emulsions in bulk (Figure 6A,D,G). Two populations of droplets were produced: one containing the n‐octanol, which floats on top of the OP water layer in the centrifuge tube, and the other one that consisted of the IP surrounded by a thin MP, which was at the bottom of the tube. We applied centrifugation at high speed (14 100 rcf) for a short time (15 s), which provided the highest yield. From the DiD intensity, it can be seen that the MP of the vesicles obtained by centrifugation was much thinner than those obtained by MP thinning in microfluidics. The spherical vesicles are again highly monodisperse (Figure 6I–K), albeit monodispersity is slightly reduced compared to before centrifugation. Further, the inner droplet retains its size during the removal of the octanol, showing that there is no appreciable loss of content due to the shear forces leading to budding. This is confirmed by the acceptor channel intensity of the FRET sensor remaining unchanged after centrifugation (Figure S12, Supporting Information). Of note is that this procedure works for both n‐octanol and n‐undecanol as oil phase equally well. Thus, centrifugation as a straightforward postproduction step provides vesicles with minimal oil. It can be performed in bulk and within 15 s, without the need for specific additives.

**Figure 7:**
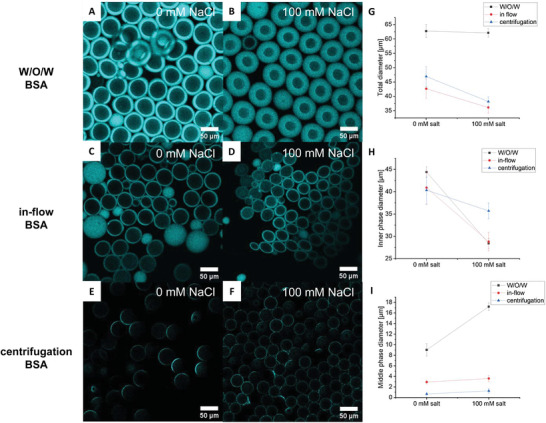
Comparison of the response of liposomes prepared with different methods upon a hyperosmotic upshift. A–F) Fluorescence confocal microscopy images of W/O/W emulsions and liposomes prepared by the in‐flow and centrifugation methods before and after an osmotic upshift with 100 mm NaCl. MP was visualized with DiD. For a visual comparison of the MP thickness: Panel (E) is from the same preparation as Panel (A), and Panel (C) is from the same device. G–I) Corresponding changes in liposome dimensions upon adding NaCl, showing dissimilar shrinkage behavior depending on the thickness of the MP layer. IP contains 80 mg mL^–1^ starting concentration BSA and the usual buffer, MP is composed of n‐octanol and lipids. Data are mean values (*n* = 50). Error bars are SD.

We find that vesicles with the thicker MPs allow higher salt‐induced shrinkage than the vesicles obtained from centrifugation (**Figure** **7**). We see that the addition of 100 mm NaCl shrinks the W/O/W and the in‐flow vesicles to the same extent, while the vesicles obtained from centrifugation only shrink marginally as judged from the diameter of the inner phase. Moreover, we find that the direct addition of 200 mm NaCl leads to significant lysis in the centrifugation‐obtained vesicles (Figure S11, Supporting Information), while vesicles from the other methods remain stable. Likely, the oil layer acts as a reservoir for the excess lipids during shrinkage in the same vein as reported previously.^[^ ^13^ ^]^ Efficient oil removal renders them susceptible to membrane defects and rupture during shrinkage. Hence, while a thin bilayer would be needed for many biochemical applications, a sizable middle phase is needed when manipulating vesicles and obtaining high crowding.

### 2.4 Obtaining Liposomes with High Macromolecular Crowding but with Minimized Oil Content

Because concentrating vesicles after centrifugation proved troublesome, we next examined to remove the oil layer from the stable W/O/W emulsions after an osmotic upshift. To monitor the macromolecular crowding during the concentration of the IP and subsequent oil removal steps, we incorporated a protein‐based macromolecular crowding sensor.^[^ ^16^ ^]^ The newest version is the crGE3.2 probe that contains monomeric enhanced green fluorescent protein (mEGFP) and mScarlet‐I as Förster Resonance Energy Transfer (FRET) donor and acceptor fluorescent proteins, respectively.^[^ ^18^ ^]^ An increase in crowding compresses the probe, leading to an increase in FRET efficiency, which is monitored by the ratio of the emission of the donor and the acceptor upon donor excitation. The functioning of the crowding sensors is independent of the fluorescent proteins that form a FRET pair,^[^ ^19^ ^]^ and we demonstrated the functioning of the crGE2.3 construct in yeast previously.^[^ ^18^ ^]^ Compared to previous generations, the advantage of this construct is that it is more red‐shifted, reducing the contribution of background fluorescence from the macromolecular crowders. A lower background fluorescence permits the use of fewer sensors and therefore reduces the contribution of the concentration‐dependent intermolecular FRET notable at high levels of shrinkage (see below). We first codon‐optimized the crGE2.3 for *Escherichia coli*, expressed it in *E. coli*, and purified it accordingly. We incorporated 50 µg mL^–1^ sensor with 80 mg mL^–1^ BSA as a crowding agent in the IP and used n‐octanol as the oil phase. Three different batches were produced: A batch (B1) contained the crowder BSA in the IP, another the crGE3.2 sensor (B2), and the third batch (B3) contained both BSA and the crGE3.2 sensor (**Table** **1**).

**Table 1:** Compositions of the three batches used with the FRET‐based crowding sensor for the crowding experiments. B1 is required for background fluorescence subtraction, B2 is the control of the sensor response in the absence of crowder, and B3 is the sensor with crowder

Batch	Inner phase (IP)	Middle phase (MP)	Outer phase (OP)
B1	10 mm NaPi, 80 mg mL^–1^ BSA	n‐octanol 5 mg mL^–1^ (POPC/POPG/cholesterol)	50 mg mL^–1^ Pluronic F‐127 10 mm NaPi
B2	10 mm NaPi, 50 µg mL^–1^ crGE3.2		
B3	10 mm NaPi, 80 mg mL^–1^ BSA, 50 µg mL^–1^ crGE3.2		

**Figure 8:**
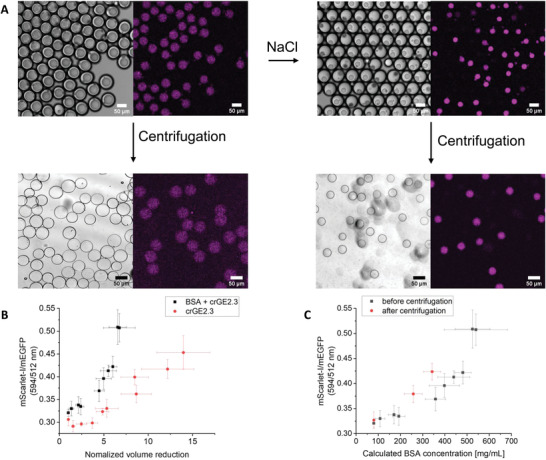
Obtaining crowded minimal‐oil liposomes in a stepwise protocol monitored with a macromolecular crowding sensor. A) Workflow and fluorescence confocal microscopy images of the crowded vesicle production. B1 and B3 are mixed. Left panels are the brightfield, and right panels are the fluorescence of the macromolecular crowding sensor crGE2.3. Excitation: 488 nm, Emission 600–700 nm. The brightfield and fluorescence images were taken with a time delay for focusing and do not perfectly overlay due to liposome movement. B) Response of the macromolecular crowding sensor before centrifugation with (B3 − B1) and without BSA (B2). The FRET/donor ratio is plotted versus the normalized shrinkage of the inner droplet, showing the contributions of BSA versus the contribution of the control conditions. C) The crGE3.2 readout before and after centrifugation plotted versus the BSA concentration in the vesicles showing no loss of crowding. The BSA concentration was calculated from the relative shrinkage of the vesicles. IP contains 80 mg mL^–1^ starting concentration BSA and the usual buffer, MP is composed of n‐octanol and lipids. Data are mean values (*n* = 10 emulsions). Error bars are SD.

B1 and B3 were mixed and imaged together, while B2 was imaged separately. B1 provided the autofluorescence of BSA needed for subtraction from B3 fluorescence. We titrated NaCl to the OP, where each concentration was performed on a new sample. We determined the emission intensity in the donor (mEGFP), the FRET (mScarlet‐I exited with mEGFP), and the acceptor (mScarlet‐I) channels, and determined the FRET/donor ratio after subtraction of the BSA background fluorescence (B1). We see that the FRET ratio increases with the NaCl concentration in the OP, as expected from an increase in macromolecular crowding due to the BSA (**Figure** **8**A,B, Figure S14, Supporting Information). The IP shrinks to the same extent as without the crowding sensor (Figure S13, Supporting Information), leading to a maximum crowding of ≈650 mg mL^–1^, corresponding to FRET ratios of ≈0.5 for each of the three independent experiments. The emulsions without crowder (B2) shrink more under the same conditions and are concentrated ≈14 times. Crowders may reduce compression by a nonlinear response of the internal osmotic pressure on the crowder concentration. Without crowder, we see an increase in FRET ratio, which may be caused by an increase in intermolecular FRET. This increase in FRET is less than with crowder. Likely, using even lower sensor concentrations would reduce the intermolecular FRET contribution. Nonetheless, it is clear that the FRET ratios in the shrunken compartments increase due to BSA, as previously shown in bulk experiments, and that the IP can therefore be crowded successfully.

Although having high crowding in the W/O/W emulsions would already benefit many applications, we next attempted to remove the oil layer to achieve highly crowded liposomes with a minimal oil membrane. As shown above, these cannot be obtained by shrinkage of the liposomes after centrifugation, which would lead to leakage and membrane rupture. In our simple two‐step protocol, we first shrank the IP to achieve the desired crowding and subsequently removed the oil phase through centrifugation (Figure 8A). In this process, the IP diameter does not change, and the concentrations and number of molecules inside should thus remain the same (Figure 6I). Applying the same centrifugation protocols for three W/O/W emulsions set at different BSA crowding levels with NaCl, we find that the resulting vesicles retain their reduced size. They are all similar regarding monodispersity and visual appearance, and the procedure was highly reproducible. Regarding the macromolecular crowding inside the generated vesicles, we see that the crGE3.2 sensor gave a similar ratio before and after removing the oil layer (Figure 8C, Figures S15 and S16, Supporting Information). These ratios correspond with the calculated BSA concentration from the IP volume, albeit that there is minor experiment‐dependent variation, possibly due to uncertainties in volume determination. We thus retain desired high levels of crowding in liposomes after removing the oil phase.

## 3 Conclusion and Discussion

Macromolecular crowding is an intrinsic property of living cells, where many functionalities are needed within a limited volume. Intracellular processes such as transcription and translation may have evolved to perform optimally in their crowded native environment. Thus, to obtain the best performance or mimic processes in the living cell for study, sensing, diagnostics, or drug screening, it is highly desirable to be able to tune macromolecular crowding inside artificial cells. Our approach here shows a two‐step protocol that allows first to tune the internal solution of the synthetic cell, after which the bilayer can be formed by effective oil removal through centrifugation.

Monodisperse artificial cells with high crowding and a minimal oil‐containing lipid bilayer have not yet been reported before. Crowded synthetic cells have been made before with capillary‐based microfluidics, where a large oil pocket that resided inside the bilayers buffers excess lipid.^[^ ^13^ ^]^ Also here, crowding was tuned by external osmolality. We made use of these stabilizing effects by retaining the oil layer when concentrating the content of the synthetic cell. However, living cells do not contain an oil pocket nor have an appreciable oil phase, and this needs to be removed to incorporate biomembrane‐type functions. Moreover, the oil will also partition into the aqueous phases and possibly interfere with a biochemical reaction. Removal of oil phases has been described by, for example, oil extraction by the addition of ethanol or by applying shear forces through a high flow that separates the oil layer based on its density.^[^ ^10^, ^11^, ^20^ ^]^ As we preferred not to add ethanol to our system, we applied high flow conditions. In contrast to previous work, we did not perform this in microfluidics during formation because we first wanted to manipulate the compartments, while we show that it does work on our system as well (Figure 6). Instead, we show that centrifugation, reminiscent of the batch‐based production of vesicles through the classical phase transfer method, produces defined and homogeneous liposomes. The separation of the production steps gave thinner bilayers in our hands and more control over the separate production steps. We believe that centrifugation could be a general and simple method to transform W/O/W emulsions into liposomes.

While the method provides a high yield of vesicles with high internal crowding, there is room for further improvement. Complete depletion of oil through dilution with OP led to debris in the sample as some vesicles collapsed. However, it was recently shown that the mixture could be cleaned using a microfluidic device, and this can be applied here as well.^[^ ^21^ ^]^ Further, the choice of oil remains crucial; while octanol is overall easier to work with as it mixes better with the lipids than higher alcohols, it has a high water partitioning coefficient and may interfere with more complex biochemical reactions. Therefore, the search for an oil phase that mixes well with lipids and a near‐zero water partitioning coefficient is still ongoing. Nonetheless, the fabrication principles discussed here would be mostly unchanged with different oils, and centrifugation of manipulated W/O/W emulsions would provide artificial cells.

The method shows great promise in studying long‐term effects in crowded compartments, such as the aging of a protein or a protein assembly, due to its high stability. Long‐term studies with similar compositions and equally sized containers would show intrinsic noise and stochastic behavior in single compartments, which is intrinsic to biology with a limited number of molecules in a cell. In addition, the ability to make bilayers with a crowded interior makes way for the study of cytosolic crowding effects on membrane proteins. These potential therapeutic targets would display more native‐like behavior in drug screening efforts and allow better study of their biological properties. Together, we foresee that the method to construct crowded artificial cells presented here will be an asset to drug screening efforts, diagnostic tool development, and for the study of biomolecules in a more relevant environment than a simple buffer.

## 4 Experimental Section

### Materials

All materials were used as purchased unless noted otherwise. 1‐heptanol (99%), 1‐octanol (99%), 1‐nonanol (98%), 1‐decanol (98%), 1‐undecanol (99%), Poly(diallyldimethylammonium chloride) (PDADMAC), poly(sodium 4‐styrenesulfonate) (PSS), hydrogen peroxide (H_2_O_2_) (30 wt%), hydrochloric acid (HCl) (37 wt%), bovine serum albumin (BSA), cholesterol, Ficoll PM70, 3‐(trimethoxysilyl)propyl acrylate (TMSPMA) (92% with 100 ppm BHT), trichloro(1H,1H,2H,2H‐perfluorooctyl)silane (PFOCTS) (97%), Pluronic F‐127 and sodium chloride (NaCl) were purchased from Sigma Aldrich. 1‐Palmitoyl‐2‐oleoyl‐sn‐glycero‐3‐phosphocholine (POPC), 1‐palmitoyl‐2‐oleoyl‐sn‐glycero‐3‐phospho‐(1’‐rac‐glycerol) (sodium salt) (POPG) was obtained from Avanti polar lipids. DiIC_18_(5) solid (1,1’‐Dioctadecyl‐3,3,3’,3’‐Tetramethylindodicarbocyanine, 4‐Chlorobenzenesulfonate Salt) (DiD) was purchased from ThermoFischer Scientific. Poly(dimethylsiloxane) (PDMS) and curing agent were obtained as SYLGARD 184 Silicone Elastomer Kit from DOW Corning.

### Expression and Purification of Crowding Sensor

The synthetic gene encoding crGE2.3, codon‐optimized for *E. coli* expression, was obtained from GeneArt. The gene was subcloned into pRSET‐A between NdeI and HindIII. The plasmid was transformed into the *E. coli* strain BL21(DE3). The cells were grown to an OD^600^ of 0.6 in LB medium with 50 µg mL^−1^ carbenicillin at 37 °C, after which the cells were induced overnight with 1 mm isopropyl‐*β*‐d‐thiogalactoside (IPTG) at 30 °C. The cells were spun down at 5000 g, 4 °C for 15 min, resuspended in lysis buffer (50 mm sodium phosphate, 500 mm NaCl, pH 7.4; 6 mm MgCl_2_, 1 µg mL^−1^ DNaseI, 1 mg mL^–1^ lysozyme and 0.2 mL EDTA‐free protease inhibitor) and lysed by high pressure homogenizer (20 000 psi, Constant Systems Ltd Muti‐Shot). The lysate was cleared by centrifugation (16 000 g, 4 °C, 30 min), supplemented with 50 mm imidazole and purified by immobilized metal ion affinity chromatography (IMAC) on HisTrap column (wash/elution buffer: 50/500 mm imidazole, 50 mm NaPi, 500 mm NaCl, pH 7.4). The sensor was then transferred in 10 mm NaPi, pH 7.4. The expression and purification were analyzed by 10% SDS‐PAGE and the bands were visualized by Coomassie staining. Fractions containing pure protein were aliquoted and stored at −80 °C.

### Microfluidic Device Fabrication

Fabrication of the PDMS‐based microfluidic device was performed as described previously.^[^ ^22^ ^]^ The positive master mold was printed on a with 3‐(trimethoxysilyl)propyl acrylate silanized microscopy glass using the dip‐in laser‐lithography method. To fully solidify the master, it was post‐cured for 12 h using ultraviolet light source (302 nm, 8 W). To render the surface of the master mold hydrophobic, it was coated with trichloro(1H,1H,2H,2H‐perfluorooctyl)silane using vapor‐phase deposition in an exicator at room temperature overnight. From this master, the microfluidic chip was produced by molding Poly(dimethylsiloxane) (DOW Corning, Sylgard 184 plus curing agent, 10:1 (w/w)) onto the master and crosslinking overnight at 60 °C in an oven. The hardened PDMS mold was cut out from the master and holes for inlets and outlet tubes were punched using a biopsy puncher (0.75 mm retractable cutting cannula from Electron Microscopy Science). The PDMS mold was washed with iso‐propanol (3 times) and demineralized water (3 times), and a glass slide (VWR Microscope Slides with cut edges, 1.0 mm thickness, 76 × 26 mm) with acetone (1 time), iso‐propanol (3 times) and demineralized water (3 times). The surface of glass slide was plasma treated by vacuum ultraviolet irradiation (TePla 100 Plasma System, PVA MPS GmbH) under an oxygen pressure of 120 Pa (40 mL min^–1^) at 200 W for 1 min, followed by a simultaneous plasma treatment of both glass slide and PDMS mold under an oxygen pressure of 120 Pa (40 mL min^–1^) at 100 W for 1 min. After treatment, the two were bonded together and placed over night in an oven at 60 °C.

### Channel Treatment

To obtain a microfluidic device with hydrophobic and hydrophilic domains, surface treatment solutions were injected into the device, rendering certain areas of the device hydrophilic. Hydrophilic surface modification of the second cross section up to the outlet is required to allow sufficient wetting of aqueous continuous solutions. The used procedure is inspired by previously described flow‐confinement techniques.^[^ ^23^ ^]^ In short, the inner aqueous phase inlet was blocked using a sealed Teflon tube. N_2_‐counterflow was added from the middle phase inlet, using a pressure pump (≈40 mbar) (membraPure: 3× Druckkammer). The treatment solutions were injected from the outlet and directed toward the second cross section. The flow was carefully adjusted by using a pressure pump (≈80 mbar) (membraPure: 3× Druckkammer). First the channels were oxidized by flushing a 2:1:1 mixture of milli‐Q water, hydrochloric acid and hydrogen peroxide into the device (5 min). Next, an aqueous solution of positive polyelectrolyte poly(diallyldimethylammonium chloride) (200–350 kDa, 5 wt% in 0.5 m NaCl) was flushed into the device (2 min). The channel was then rinsed by flushing with ultra‐pure water (1 min) and lastly, an aqueous solution of the negative polyelectrolyte poly(sodium 4‐styrenesulfonate) (70 kDa, 2 wt% in 0.5 m NaCl) was flushed into the device (2 min).

### W/O/W Droplet Formation

The W/O/W double emulsion was prepared using an IP solution of sodium phosphate (NaPi) (10 mm), the respective crowder BSA (80 mg mL^–1^) or Ficoll PM70 (80 mg mL^–1^) and in experiments in which crowding was to be characterized, the crowding sensor crGE2.3 (50 µg mL^–1^) was included to the previously mentioned components. The MP consisted of a lipid blend solution of POPC:POPG:cholesterol (5 mg mL^–1^) in a ratio of 8.5:1.0:0.5 and the fluorescent dye 1,1’‐Dioctadecyl‐3,3,3’,3'‐Tetramethylindodicarbocyanine, 4‐Chlorobenzenesulfonate salt (0.1 mol%) dissolved in 1‐heptanol, 1‐octanol, 1‐nonanol, 1‐decanol, or 1‐undecanol. The OP solution is a mixture of sodium phosphate (10 mm) and Pluronic F‐127 (50 mg mL^–1^). The solutions were introduced into the respective inlets by loading them into a glass syringe (1 mL; Hamilton Glass Syringe) with needle (0.5 × 16 mm; BD Microlance 3) and Teflon tube (Outer Diameter: 0.9 mm, Inner Diameter: 0.4 mm; Techlab GmbH) and injecting them using a pump (Harvard Apparatus P11). The flow rates were adjusted as required for a successful W/O/W emulsion production. Typical values for the W/O/W production were 500–700 µL h^–1^ for the OP, 160–200 µL h^–1^ for the MP and 50–70 µL h^–1^ for the IP. For the inflow method the flow rate of the OP was increased until a splitting of excess MP at the second cross‐junction was observed. The W/O/W emulsion was collected from the outlet into an Eppendorf tube (1.5 mL; Eppendorf Tubes) prefilled with OP solution (0.3 mL) using a Teflon tube.

### Oil Removal by Centrifugation

For the removal of the MP from the W/O/W double emulsions by centrifugation, the OP solution (0.3 mL) in which the W/O/W droplets were stored and the W/O/W droplets (0.3 mL) were transferred into a new Eppendorf tube (1.5 mL; Eppendorf Tubes). They were then centrifuged (14 100 rcf, 15 s) and could be collected from the bottom of the Eppendorf tube.

### Oil Removal by Pinch‐Off in Microfluidic Device

The injection of all fluids into the device was conducted using a pump (Harvard Apparatus P11), glass syringe (1 mL; Hamilton Glass Syringe) with needle (0.5 × 16 mm; D Microlance 3) and Teflon tube (Outer Diameter: 0.9 mm, Inner Diameter: 0.4 mm; Techlab GmbH). First, the trapping device was filled with OP solution by injecting it (50 µL h^–1^) into the OP inlet until completely filled and all air was removed. After turning of the OP flow, the syringe containing the W/O/W was connected to the W/O/W inlet and the third inlet closed off by using a short Teflon tube filled with glue. The W/O/W double emulsion was then injected (5 µL h^–1^) into the device using the W/O/W inlet. Once enough W/O/W was trapped, the W/O/W flow was stopped. To induce the pinch‐off the OP flow rate was turned back on (50 µL h^–1^) until the removal of the oil was complete.

### W/O/W Emulsion Crowding

For all crowding experiments, except for the crowding experiments with the crowding sensor (crGE2.3), were done on glass slides (VWR Microscope Slides with cut edges, 1.0 mm thickness, 76 × 26 mm). The W/O/W emulsions, in‐flow emulsions, or vesicles (20 µL) were added onto a glass slide and immediately after the NaCl(aq.) solution was added. After 5 min the crowding was observed and characterized. For the W/O/W crowding experiments with crowding sensor (crGE2.3), an Eppendorf (1.5 mL; Eppendorf Tubes) was filled with the aqueous NaCl solution (100 µL) with double the concentration of the targeted final concentration. The W/O/W emulsion (100 µL) was added and after 5 min the crowded W/O/W emulsion could be characterized. The procedure for the centrifuged WO/W crowding experiments with crowding sensor was identical, except that for both salt solution and W/O/W emulsion 300 µL instead of 100 µL, respectively, was used. After 5 min, the crowded W/O/W emulsion was centrifuged (14 100 rcf, 15 s) and could be collected from the bottom of the Eppendorf tube.

### Confocal Fluorescence Microscopy

The produced W/O/W emulsions and centrifuged W/O/W were added onto glass slides (VWR Microscope Slides with cut edges, 1.0 mm thickness, 76 × 26 mm). W/O/W emulsions and centrifuged W/O/W emulsions were imaged using confocal microscopy (Leica TCS SP8, Leica Microsystems Inc. and Leica TCS SP8 STED 3×, Leica Microsystems Inc.). The crowding sensor crGE2.3 was excited at 488 nm and the emission was split into a 510–525 nm channel for the donor detection and a 600–700 nm channel for the FRET detection. For the acceptor excitation a wavelength of 561 nm was used and a 600–700 nm channel was used for emission detection. DiD was excited at a wavelength of 633 nm and for emission a 650–720 nm channel was used. All data were analyzed using ImageJ.

### Statistical Analysis

Data points were shown as mean with error bars indicating standard deviation. The sample sizes *n* were specified in figure captions. Duplicate and triplicate measurements are provided in the Supporting Information where applicable.

## Conflict of Interest

The authors declare no conflict of interest.

## Supporting information

Supporting Information

Supplemental Video 1

Supplemental Video 2

Supplemental Video 3

## Data Availability

The data that support the findings of this study are available from the corresponding author upon reasonable request.
