# Supplementary material for: High Macromolecular Crowding in Liposomes from Microfluidics
Source: Adv Sci (Weinh). 2022 Jul 29;9(27):2201169. doi: 10.1002/advs.202201169 (PMC9507340; doi:10.1002/advs.202201169)
Supplement: Supplementary file 1 — Supporting Information [file ADVS-9-2201169-s003.pdf]

ADVANCED SCIENCE
Open Access

Supporting Information

for *Adv. Sci.*, DOI 10.1002/advs.202201169

High Macromolecular Crowding in Liposomes from Microfluidics

*Luis P. B. Guerzoni, André V. C. de Goes, Milara Kalacheva, Jakub Haduła, Matthias Mork, Laura De Laporte and Arnold J. Boersma**

Supporting information

High macromolecular crowding in liposomes from microfluidics

Luis P. B. Guerzoni[1], *André V. C. de Goes*[1,2], *Milara Kalacheva,*[1,2] *Jakub Haduła,*[1,2] *Matthias Mork,* [1,2] *Laura De Laporte,* [1,2,3] *Arnold J. Boersma*[1]*

1. DWI-Leibniz Institute for Interactive Materials, Forckenbeckstrasse 50, 52074 Aachen, Germany
2. Institute of Technical and Macromolecular Chemistry, RWTH Aachen University, Worringerweg 1, 52074, Aachen, Germany.
3. Department Advanced Materials for Biomedicine, Institute of Applied Medical Engineering, University Hospital RWTH Aachen, Forckenbeckstrasse 55, 52074 Aachen, Germany

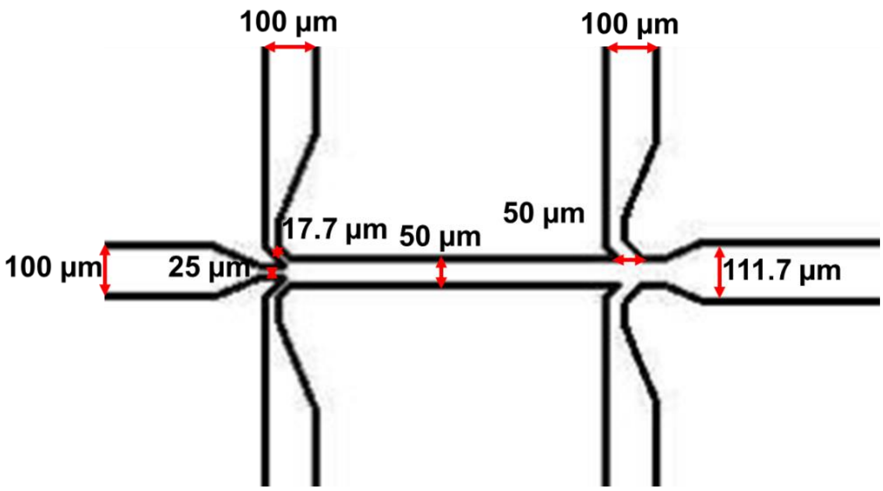

Supplementary Figure 1. Section of the microfluidic device design used to produce W/O/W emulsions showing the dimensions.

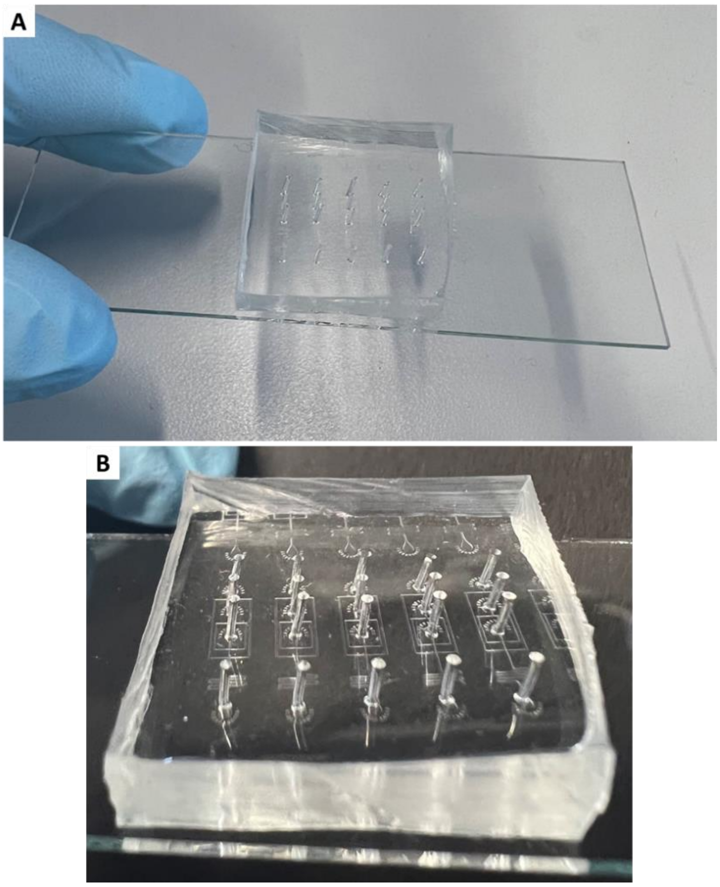

Supplementary Figure 2. Photograph of microfluidic device bonded to a glass slide.

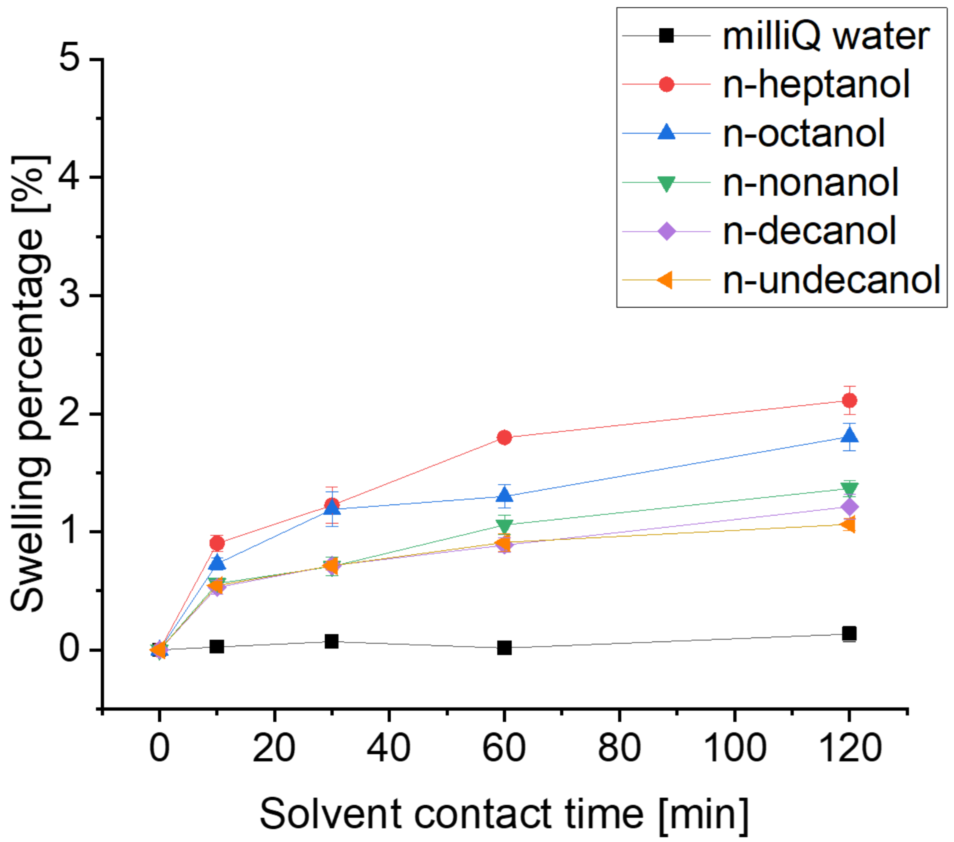

Supplementary Figure 3. Plots of swelling percentage against the solvent contact time for all tested oil phase solvents showing no significant swelling for all solvents. Shorter -CH_2- chains resulted in slightly higher swelling. Poly(dimethylsiloxane) (DOW Corning, Sylgard® 184 plus curing agent, 10:1 (w/w)) was molded onto a petri dish so that the PDMS height was leveled

at 5 mm. From the hardened PDMS, cubes (5x10x10 mm) were cut out and using a biopsy puncher, a hole was punched through the 5 mm thick side of the cube, and the cubes were weighted. A Polyethylene tube (4 cm) was inserted into the hole so that it did not appear on the other side and the whole cube could be held in the air by the tube. The tip of the tube was marked using a piece of tape, so that all cubes could be differentiated from another. A glass beaker (100 mL) was filled halfway with the respective fluid and 12 cubes were simultaneously dipped into the fluid so that the whole body was in contact with the solvent and the tube was sticking upward out of the fluid. Using the tape that was placed at the tip of the tube, the cubes were held in the fluid. After 10, 30, 60 and 120 minutes three cubes were taken out of the fluid simultaneously and after removing fluid from the surface using nitrogen gas and tissue, the cubes were immediately weighted without the tube. Using the weight before and after contact with fluid, the swelling percentage was calculated for each respective time and cube. The positive control was hexane known to give a high swelling, which gave 56.2+/-1.3% swelling. The mean swelling of the three cubes that were taken out of the solution simultaneously at the respective times was calculated and plotted against the time

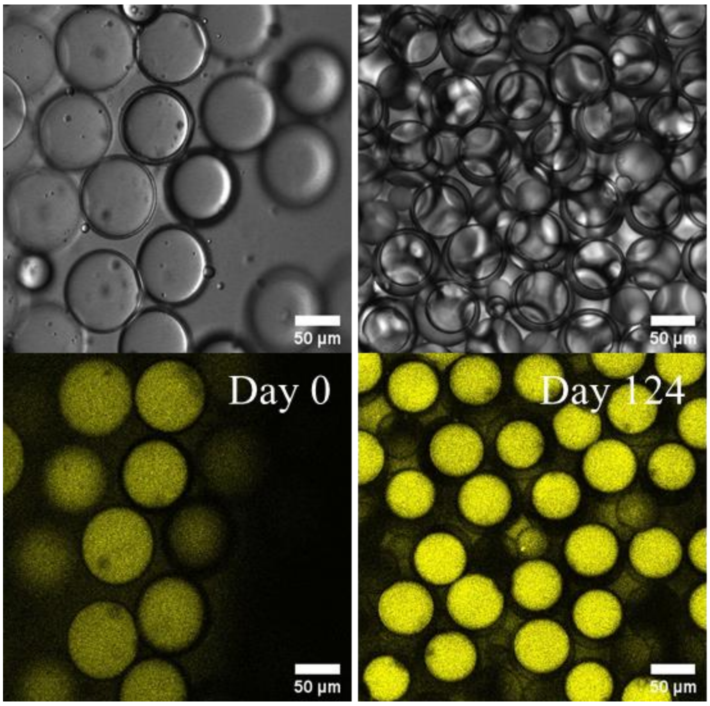

Supplementary Figure 4. W/O/W emulsions containing 150 mg/mL Ficoll PM70 are highly stable in time. Top: brightfield images, bottom: fluorescence confocal images of mVenus-mCherry containing emulsions. Excited at 488 nm, emission at 505-555 nm. the MPs contain 5 mg mL^{-1} of POPC:POPG:cholesterol 8.5/1.0/0.5 in n-octanol. The IP contains 10 mM NaPi, pH 7.4, 150 mg mL^{-1} Ficoll PM70, the OP contains 50 mg mL^{-1} Pluronic F-127 + 10 mg mL^{-1} NaPi, pH 7.4.

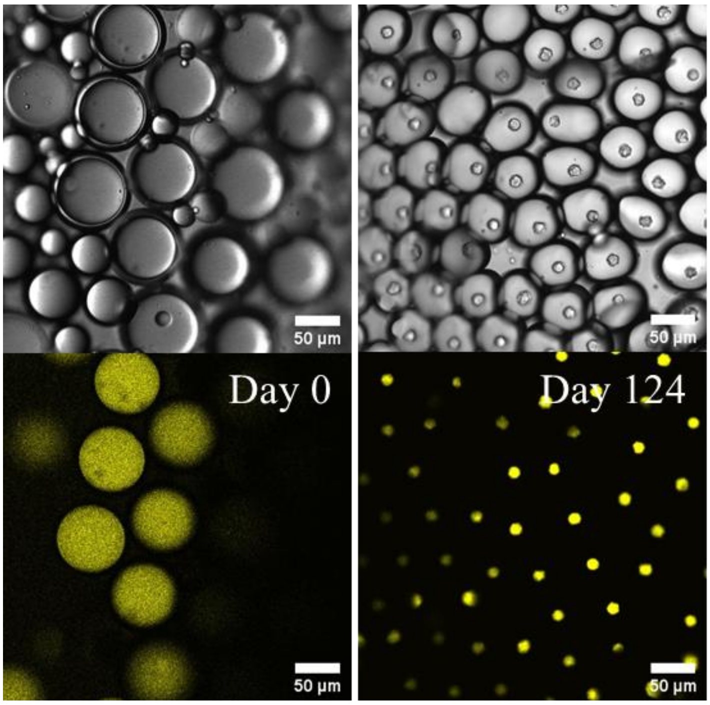

Supplementary Figure 5. W/O/W emulsions without crowder showing a significant shrinkage over time. Top: brightfield images, bottom: fluorescence confocal images of mVenus-mCherry containing emulsions. Excited at 488 nm, emission at 505-555 nm. the MPs contain 5 mg mL^{-1} of POPC:POPG:cholesterol 8.5/1.0/0.5 in n-octanol. The IP contains 10 mM NaPi, pH 7.4, the OP contains 50 mg mL^{-1} Pluronic F-127 + 10 mg mL^{-1} NaPi, pH 7.4.

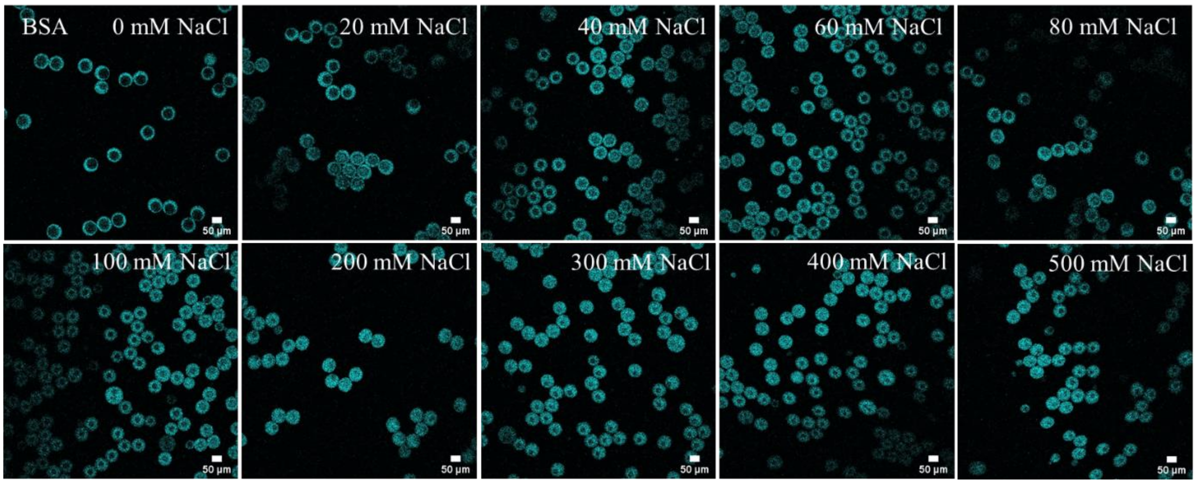

Supplementary Figure 6. Fluorescence confocal microscopy images of the W/O/W emulsions containing an 80 mg mL^{-1} starting concentration of BSA and the subsequent shrinkage upon titration of NaCl to the medium. The MP consists of n-octanol with 5 mg mL^{-1} of POPC:POPG:cholesterol 8.5/1.0/0.5. The IP contains 10 mM NaPi, pH 7.4, the OP contains 50 mg mL^{-1} Pluronic F-127 + 10 mg mL^{-1} NaPi, pH 7.4. The MP is visualized using DiD.

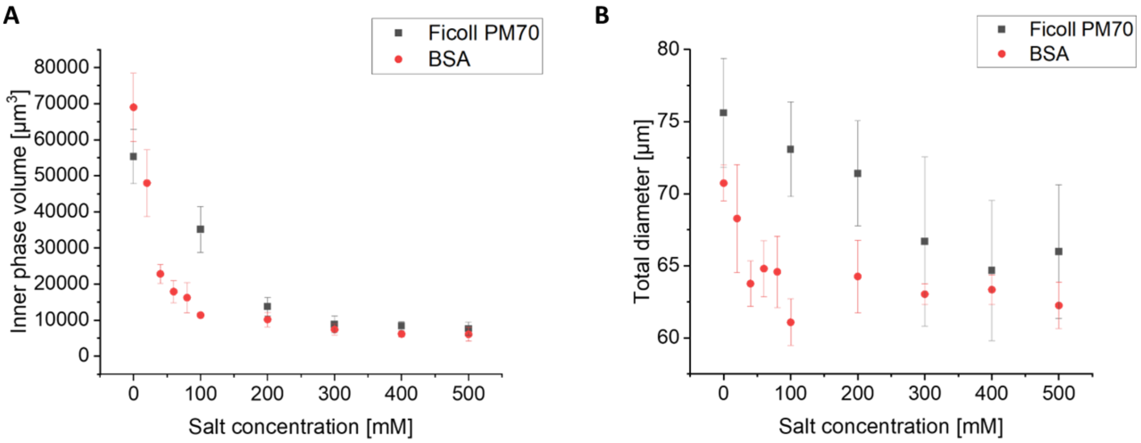

Supplementary Figure 7. A. Volume of the inner phase as function of external NaCl concentration showing that the volume shrinks with increasing salt concentration. B. Total emulsion volume (IP+MP) as function of external NaCl concentration. The MP consists of n-octanol with 5 mg mL^{-1} of POPC:POPG:cholesterol 8.5/1.0/0.5 and 0.1 mol% DiD. The IP contains 10 mM NaPi, pH 7.4, the OP contains 50 mg mL^{-1} Pluronic F-127 + 10 mg mL^{-1} NaPi, pH 7.4. Error bars are SD (n = 10 emulsions)

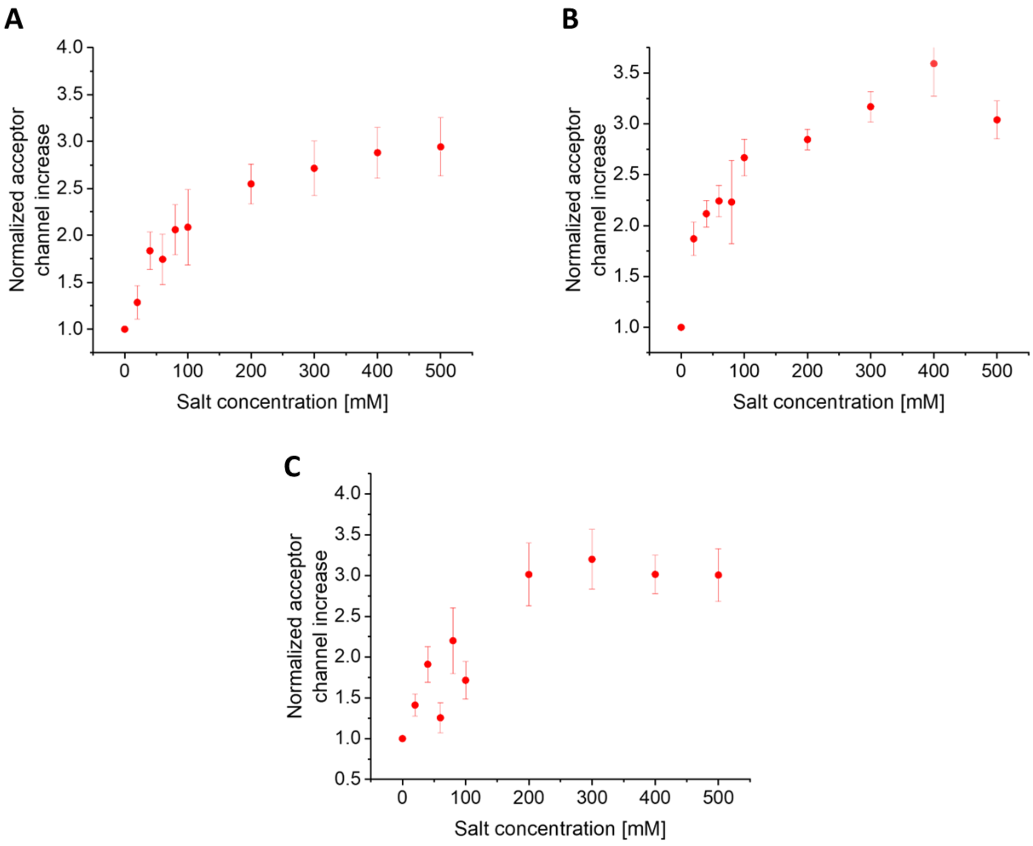

Supplementary Figure 8. Measurement of normalized acceptor channel intensity of W/O/W containing a starting concentration of 80 mg mL^{-1} BSA and subsequent intensity increase upon NaCl-induced shrinkage. The increase is less than expected from the volume, possibly due to quenching effects at high sensor concentration. A-C: Measurement performed in triplicate. The MP consists of n-octanol with 5 mg mL^{-1} of POPC:POPG:cholesterol

8.5/1.0/0.5. The IP contains 10 mM NaPi, pH 7.4, the OP contains 50 mg mL^{-1} Pluronic F-127 + 10 mg mL^{-1} NaPi, pH 7.4. Error bars are SD (n = 10 emulsions).

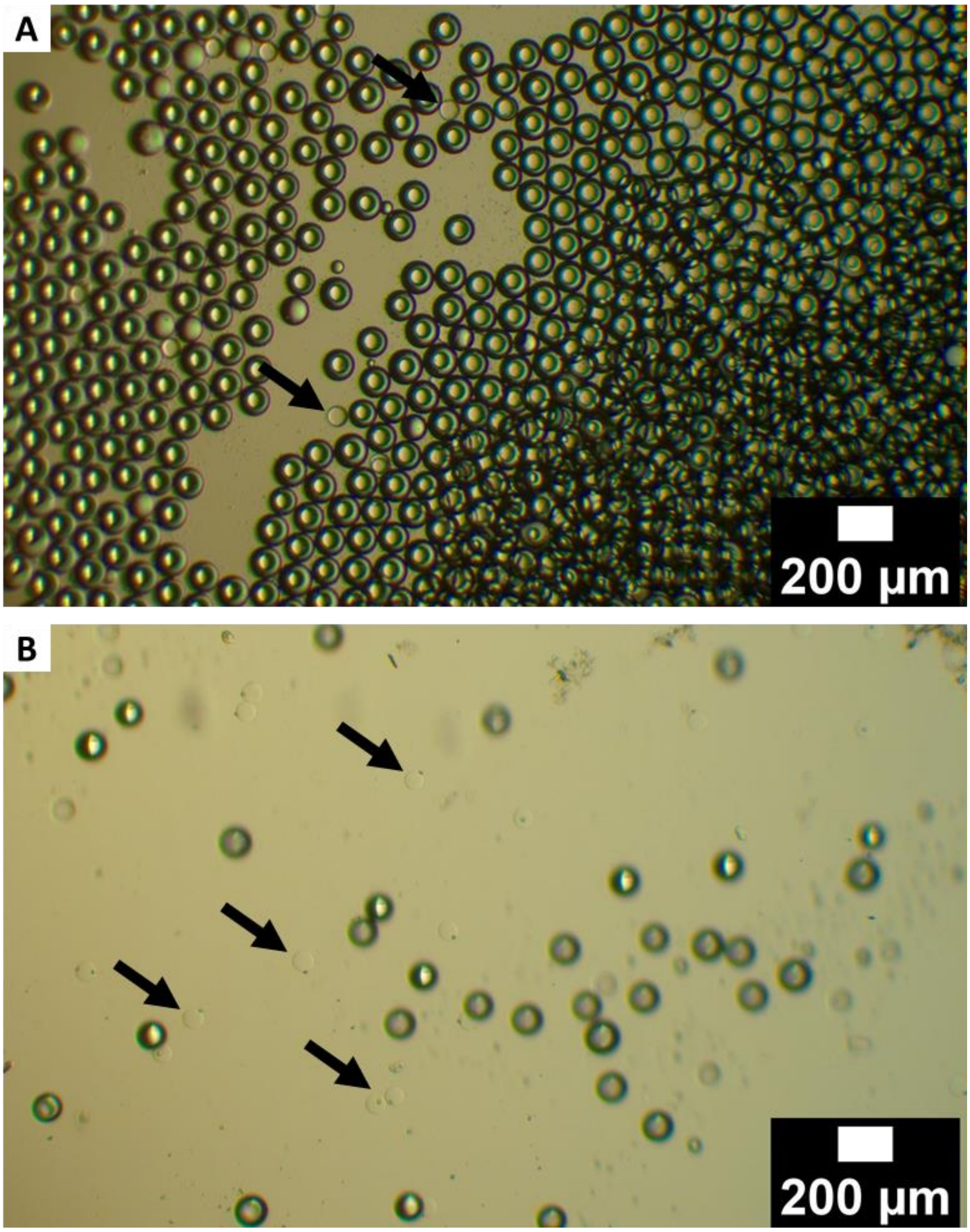

Supplementary Figure 9. Microscope images of spontaneous dewetting of undecanol after 10 days incubation at room temperature in a sealed Eppendorf tube. A. Sample collected from the top of the Eppendorf tube showing mostly W/O/W emulsions and only few IP without MP. B. Sample collected from the bottom of the Eppendorf tube with multiple liposomes. Some W/O/W emulsions are present because of imperfect sample collection. The IP consist of 80 mg mL^{-1} Ficoll PM70 and 10 mM NaPi, pH 7.4. The MP consists of n-undecanol with 5 mg mL^{-1} POPC:POPG:cholesterol 8.5/1.0/0.5. The OP contains 50 mg/mL Pluronic F-127 and 10 mg mL^{-1} NaPi, pH 7.4. Black arrows denote liposomes.

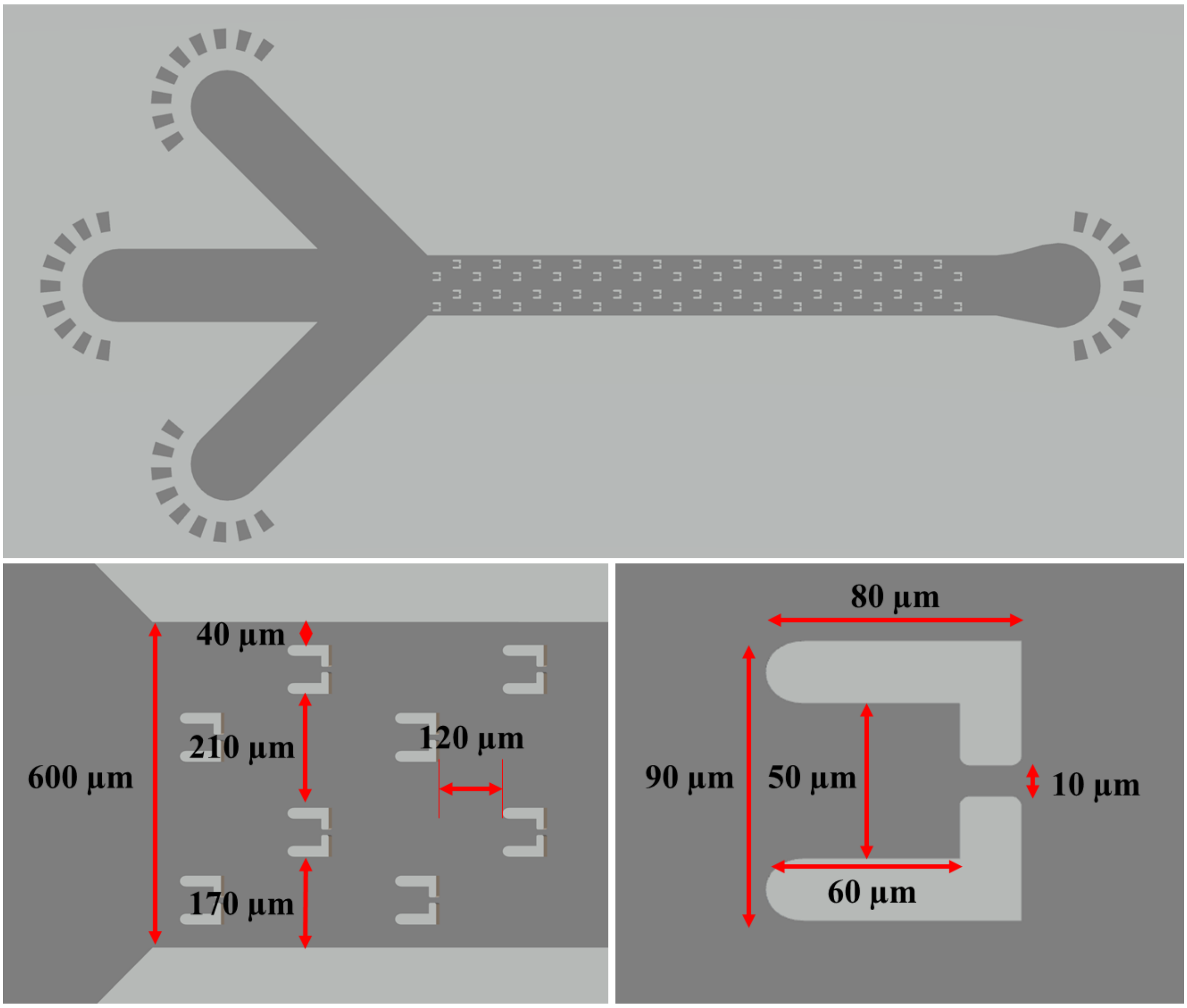

Supplementary Figure 10. Design of microfluidic device used to produce W/O/W emulsions showing the dimensions. The device has three inlets for W/O/W emulsion, OP and salt solution. The later allows for a shrinking of the IP by osmotic stress.

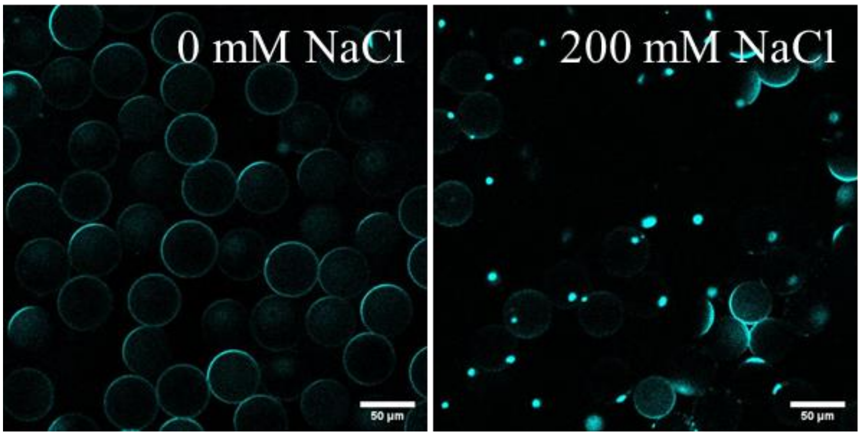

Supplementary Figure 11. Fluorescence confocal microscopy images of liposomes incorporated with Ficoll PM70 and prepared by the centrifugation method before and after an osmotic upshift with 200 mM NaCl. The IP consist of 80 mg mL^{-1} Ficoll PM70 and 10 mM NaPi, pH 7.4. The MP consists of n-octanol with 5 mg/mL POPC:POPG:cholesterol 8.5/1.0/0.5. The OP contains 50 mg mL^{-1} Pluronic F-127 and 10 mg mL^{-1} NaPi, pH 7.4. MP was visualized with DiD.

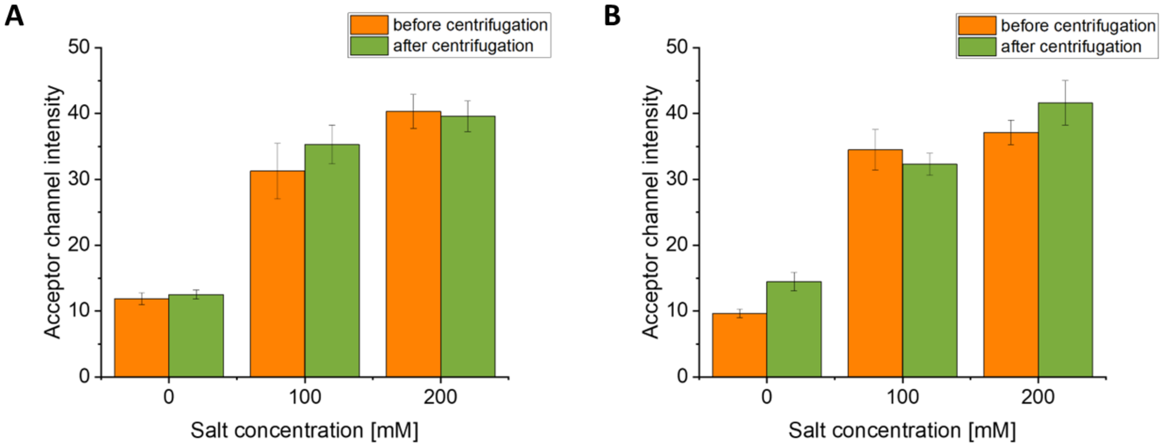

Supplementary Figure 12. Comparison of average acceptor channel intensity of the FRET sensor before and after centrifugation in duplicate. The comparison shows a similar intensity and therefore no leakage of the FRET sensor. Error bars are SD (n = 10).

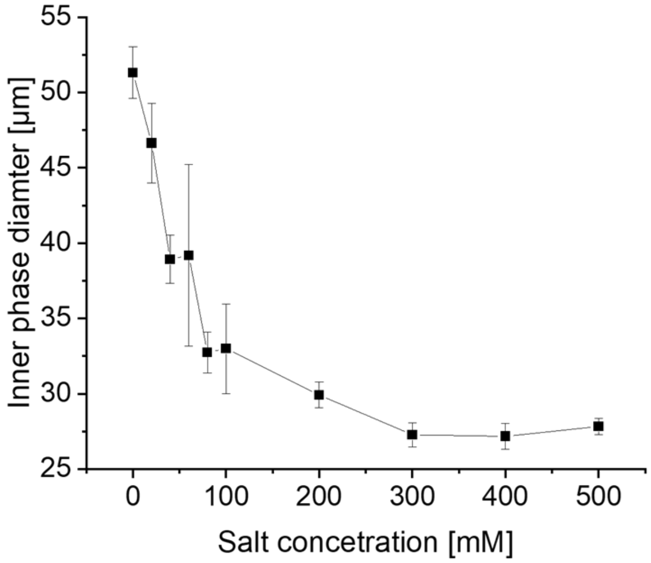

Supplementary Figure 13. Dependence of the IP diameter on the external NaCl concentration calculated from the mean IP diameter. Average over three independent crowding experiments with BSA and crGE2.3. The MP consists of n-octanol with 5 mg mL^{-1} of POPC:POPG:cholesterol 8.5/1.0/0.5 and 0.1 mol% DiD. The IP contains 10 mM NaPi, pH 7.4, the OP contains 50 mg mL^{-1} Pluronic F-127 + 10 mg mL^{-1} NaPi, pH 7.4. Average of three independent measurements, error bars are SD.

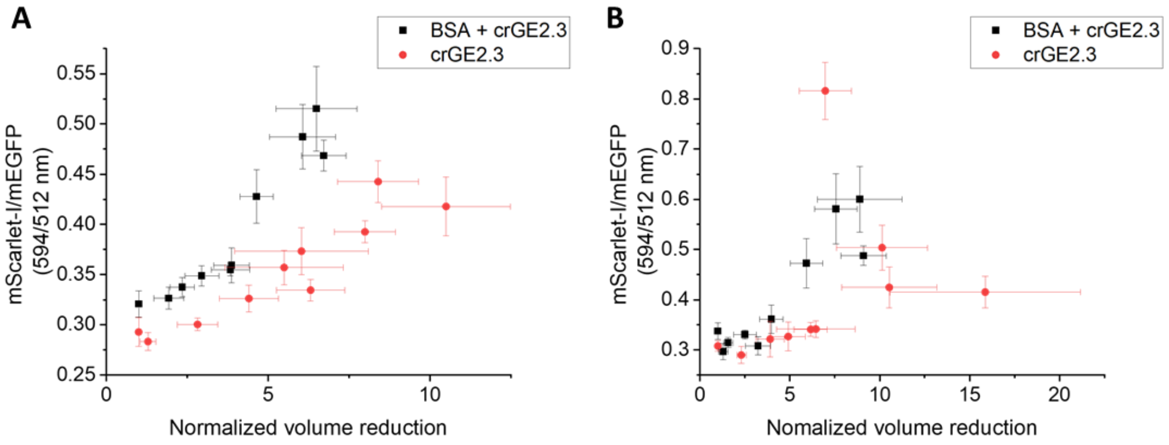

Supplementary Figure 14. Duplicate (A) and triplicate (B) measurement of the response of the macromolecular crowding sensor before centrifugation with and without BSA related to Figure 8B. The FRET/donor ratio is plotted versus the normalized shrinkage of the inner droplet, showing the contributions of BSA versus the contribution of the control conditions. IP contains 80 mg mL^{-1} starting concentration BSA and 10 mM NaPi, pH 7.4. MP is composed of n-octanol with 5 mg mL^{-1} POPC:POPG:cholesterol 8.5/1.0/0.5. OP contains 10 mM NaPi, pH 7.4, and Pluronic F-127. Error bars are SD (n = 10).

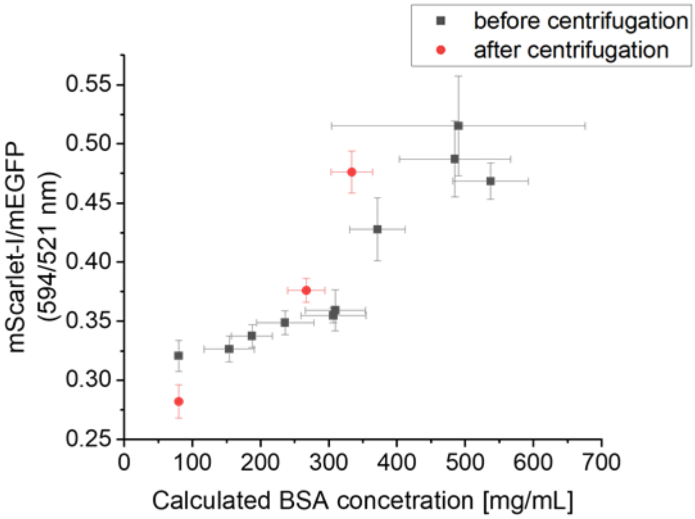

Supplementary Figure 15. The crGE3.2 readout for the second experiment before and after centrifugation plotted versus the BSA concentration in the vesicles showing no loss of crowding. The BSA concentration was calculated from the relative shrinkage of the vesicles. IP contains 80 mg mL^{-1} starting concentration BSA and 10 mM NaPi, pH 7.4. MP is composed of n-octanol with 5 mg mL^{-1} POPC:POPG:cholesterol 8.5/1.0/0.5. OP contains 10 mM NaPi, pH 7.4, and Pluronic F-127. Error bars are SD (n = 10).

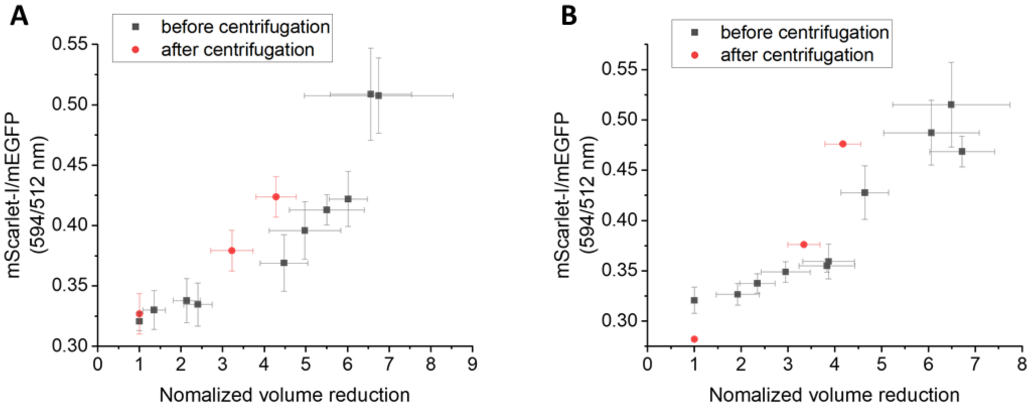

Supplementary Figure 16. The crGE3.2 readout before and after centrifugation plotted versus the normalized shrinkage of the inner droplet. A. First Experiment. B. Second Experiment. IP contains 80 mg mL^{-1} starting concentration BSA and 10 mM NaPi, pH 7.4. MP is composed of n-octanol with 5 mg mL^{-1} POPC:POPG:cholesterol 8.5/1.0/0.5. OP contains 10 mM NaPi, pH 7.4, and Pluronic F-127. Error bars are SD (n = 10).

DNA sequence *E. coli* codon-optimized crowding sensor crGE2.3

CATATGGGTCATCATCATCACCATCACAAAGGTGAAGAACTGTTTACCGGTGTTG
TTCCGATTCTGGTTGAACTGGATGGTGACGTTAATGGTCACAAATTTTCAGTTAGC
GGTGAAGGCGAAGGTGATGCAACCTATGGTAAACTGACCCTGAAATTTATCTGTA
CCACCGGCAAACTGCCGGTTCCGTGGCCGACACTGGTTACCACACTGACCTATGG
TGTTCAGTGTTTTAGCCGTTATCCTGATCACATGAAACAGCACGATTTTTTCAAAA
GCGCAATGCCGGAAGGTTATGTTCAAGAACGTACCATCTTCTTCAAAGATGACGG
CAACTATAAAACCCGTGCCGAAGTTAAATTTGAAGGTGATACCCTGGTGAATCGC
ATTGAACTGAAAGGCATCGATTTTAAAGAGGATGGTAATATCCTGGGCCACAAAC
TGGAATATAATTATAATAGCCACAACGTGTACATCATGGCCGACAAACAGAAAA
ATGGCATCAAAGTGAACTTCAAGATCCGCCATAATATTGAAGATGGTTCAGTTCA
GCTGGCCGATCATTATCAGCAGAATACCCCGATTGGTGATGGTCCGGTTCTGCTG
CCGGATAATCATTATCTGAGCACCCAGAGCAAACTGAGCAAAGATCCGAATGAA
AAACGCGATCACATGGTGCTGCTGGAATTTGTTACCGCAGCAGGTATTACCTTAG
GTATGGATGAACTGTATAAAGGATCCGGTGGTAGCGGTGGTTCAGGTGGTAGTGG
CGGTAGTGGTGGCAGCGGTGCAGAAGCAGCAGCAAAAGAAGCCGCTGCCAAAGA
AGCGGCAGCGAAAGAGGCTGCCGCAAAAGAGGCAGCAGCGAAAGAAGCAGCGG
CTAAAGCAGGTTCAGGCGGAAGCGGAGGCAGTGGTGGATCAGGCGGATCTGGTG
GCTCAGGTGCCGAGGCAGCAGCAAAAGAGGCAGCTGCTAAAGAGGCTGCTGCAA
AAGAAGCAGCCGCAAAAGAGGCAGCGGCAAAAGAAGCCGCAGCAAAAGCAGGT
AGTGGTGGAAGTGGCGGTTCCGGTGGCTCTGGTGGAAGCGGTGGCTCCGGAGTTA
GTAAAGGCGAAGCAGTTATTAAAGAATTTATGCGCTTCAAAGTGCACATGGAAG
GTAGCATGAATGGCCATGAATTTGAAATCGAAGGTGAAGGTGAGGGTCGTCCGT
ATGAAGGCACCCAGACCGCAAAACTGAAAGTTACCAAAGGTGGTCCGCTGCCGT
TTAGCTGGGATATTCTGAGTCCGCAGTTTATGTATGGTAGCCGTGCATTTATCAAA
CATCCGGCAGATATCCCGGATTATTACAAACAGAGCTTTCCCGAAGGTTTTAAAT
GGGAACGTGTGATGAATTTTGAGGATGGTGGTGCAGTTACCGTTACACAGGATAC
CAGCCTGGAAGATGGCACCCTGATCTATAAAGTTAAACTGCGTGGCACCAATTTT
CCGCCAGATGGTCCTGTTATGCAGAAAAAAACCATGGGTTGGGAAGCAAGCACC
GAACGTCTGTATCCTGAAGATGGCGTTCTGAAAGGTGATATCAAAATGGCACTGC
GTCTGAAAGATGGTGGTCGTTATCTGGCAGATTTCAAAACCACCTACAAAGCCAA
AAAACCGGTTCAGATGCCTGGTGCATATAATGTTGATCGCAAACTGGATATCACC
AGCCATAATGAAGATTATACCGTGGTGGAACAGTATGAACGTAGCGAAGGTCGT
CATAGTACCGGTGGCATGGATGAATTATACAAAGGTGGCACCTAAAAGCTT
